# Strategic Design and Synthesis of Ferrocene Linked Porous Organic Frameworks toward Tunable CO_2_ Capture and Energy Storage

**DOI:** 10.3390/ijms241512371

**Published:** 2023-08-02

**Authors:** Aya Osama Mousa, Cheng-Hsin Chuang, Shiao-Wei Kuo, Mohamed Gamal Mohamed

**Affiliations:** 1 Department of Materials and Optoelectronic Science, Center of Crystal Research, National Sun Yat-sen University, Kaohsiung 804, Taiwan; aya623514@gmail.com; 2 Institute of Medical Science and Technology, National Sun Yat-sen University, Kaohsiung 804201, Taiwan; chchuang@imst.nsysu.edu.tw; 3 Department of Medicinal and Applied Chemistry, Kaohsiung Medical University, Kaohsiung 807, Taiwan; 4 Chemistry Department, Faculty of Science, Assiut University, Assiut 71516, Egypt

**Keywords:** ferrocene, 6,6′-(1,4-phenylene)bis(1,3,5-triazine-2,4-diamine, condensation reaction, porosity, CO_2_ capacity, supercapacitor

## Abstract

This work focuses on porous organic polymers (POPs), which have gained significant global attention for their potential in energy storage and carbon dioxide (CO_2_) capture. The study introduces the development of two novel porous organic polymers, namely FEC-Mel and FEC-PBDT POPs, constructed using a simple method based on the ferrocene unit (FEC) combined with melamine (Mel) and 6,6′-(1,4-phenylene)bis(1,3,5-triazine-2,4-diamine) (PBDT). The synthesis involved the condensation reaction between ferrocenecarboxaldehyde monomer (FEC-CHO) and the respective aryl amines. Several analytical methods were employed to investigate the physical characteristics, chemical structure, morphology, and potential applications of these porous materials. Through thermogravimetric analysis (TGA), it was observed that both FEC-Mel and FEC-PBDT POPs exhibited exceptional thermal stability. FEC-Mel POP displayed a higher surface area and porosity, measuring 556 m^2^ g^−1^ and 1.26 cm^3^ g^−1^, respectively. These FEC-POPs possess large surface areas, making them promising materials for applications such as supercapacitor (SC) electrodes and gas adsorption. With 82 F g^−1^ of specific capacitance at 0.5 A g^−1^, the FEC-PBDT POP electrode has exceptional electrochemical characteristics. In addition, the FEC-Mel POP showed remarkable CO_2_ absorption capabilities, with 1.34 and 1.75 mmol g^−1^ (determined at 298 and 273 K; respectively). The potential of the FEC-POPs created in this work for CO_2_ capacity and electrical testing are highlighted by these results.

## 1. Introduction

Numerous energy-collecting techniques have been developed as a result of the rising need for energy that emits no greenhouse gases. Although they are essential, renewable energy sources cannot provide all of our daily energy needs. Investigating reasonable and cost-effective methods of energy collection and storage is therefore important. Due to their excellent qualities including high energy densities, quick charge/discharge rates, and extended lifespan, supercapacitors (SCs) have received a lot of interest in this respect [1,2,3]. SCs are appropriate for a variety of applications, including biological defibrillators and wind turbines, thanks to their advantageous characteristics [4,5,6]. A number of factors, such as (i) reactions occurring at the surfaces of the electrode materials, (ii) physical charge separation across the EDLC surfaces, frequently using porous carbons as electrodes, and (iii) faradaic reactions involving organic molecules with redox activity and electrolytes, all have an impact on the ability of SCs to store electrical charge. The characteristics of the electrode material, including its composition, have a significant influence on how well SCs work as a consequence [7,8]. High nitrogen content, large electrolyte-accessible surface areas, and hierarchical porous structures are all qualities that electrode materials should have in order to satisfy the needs of energy storage applications [9,10]. These features enable enhanced energy storage capacity in supercapacitors. Various compounds, including metal oxide and porous carbonaceous precursors, are frequently utilized as electrode materials in supercapacitors. Electrode materials used in supercapacitors typically consist of a diverse range of substances, including metal oxides, porous carbonaceous precursors, hydroxides, and sulfides. However, to address concerns regarding hazardous inorganic chemicals, there is growing interest in exploring organic electroactive materials as potential alternatives [11,12,13,14]. The environmental friendliness, versatility, and accessibility to raw resources are key advantages of these organic materials. In the context of supercapacitors, electrochemical reactions primarily occur on the electrode’s surface, while the pores within the electrode facilitate ion mitigation. This division of roles allows for efficient charge storage and rapid charge/discharge rates in the supercapacitor system. Anthropogenic carbon dioxide (CO_2_) emissions have significant implications for human existence and the environment, leading to increased global temperatures and altered weather patterns [15,16]. Consequently, there is a pressing need to reduce emissions and CO_2_ levels through sequestration and capture methods, which has become a critical focus for both academic research and industry. The conventional wet cleaning technique, involving the use of aqueous solutions of alkanolamine (known as amine scrubbing), has been widely employed for CO_2_ capture and separation. However, this method has several drawbacks. It exhibits high energy consumption, is costly to implement, has limited efficiency, poses challenges with solvent renewal, results in solvent loss, and leads to equipment corrosion due to the presence of amines [17,18,19]. To address these limitations, new and efficient strategies are required to enhance CO_2_ capture in industrial operations while simultaneously reducing emissions. Over the past decade, due to their exceptional characteristics, POPs have attracted substantial attention in both the academic and industrial worlds. These characteristics include high surface areas, suitable pore sizes, excellent thermal stability, low density, diverse composition, and low regeneration energy requirements [20,21]. Due to POPs’ special properties, a variety of fascinating prospective uses have become available such as biomedicine, sensors, gas capture and separation, photovoltaics, optical devices, and hydrogen evolution [22,23,24,25,26,27,28,29,30,31,32]. Given the challenges posed by energy shortages, global warming, and carbon dioxide (CO_2_) emissions, there is a growing need to focus on CO_2_ capture and supercapacitors (SCs). POPs have become desirable candidates for CO_2_ uptake and as active electrode materials in SCs because of their unique characteristics [33,34,35]. Studies by Jiang et al. have shown that POPs exhibit a high capacity for absorbing CO_2_ or I_2_ [36,37]. Various POP structures incorporating covalently linked linkages, such as boroxine, triazine, hydrazine, imide, and imine units, have been synthesized using chemical reactions like Suzuki, Sonogashira, Schiff base formation, Heck, Yamamoto, and Friedel couplings [38,39,40,41,42,43,44]. POPs can be categorized into several materials, including COFs, CMPs, PIMs, HCPs, and CTFs [45,46,47,48]. These diverse categories demonstrate the versatility and potential of POPs for various applications, highlighting their importance in the development of advanced materials for addressing environmental concerns and energy-related challenges. FEC has gained significant attention due to its stable sandwich-like structure, high electron density, and redox abilities [49]. There are new opportunities for enantioselective catalysis, redox batteries, sensing properties, and magnetic switches when a ferrocenyl unit is included in a polymer framework [50,51,52,53]. Molecular techniques utilizing ferrocene-based polymers have rapidly expanded in fields such as gas sorption, redox batteries, pollution removal, precursor-derived ceramics, catalysis, and memory devices [54,55,56,57,58]. For example, the FcTz-POP exhibited significantly higher iodine capture (396 wt% at 348 K) compared to a similar ferrocene-free BpTz-POP, with an increase of 1.8 times [59]. Ma and colleagues developed Fc-based CMPs with Fc linkages for excellent performance in degrading methylene blue (99%) under visible light [60]. Additionally, Tan et al. synthesized FcCMP-1 and FcCMP-2 for dye removal, which had good gas storage capacity [61]. Furthermore, Samy et al. successfully synthesized BP-FC-CMP with a capacitance of 608 F g^−1^ [62]. In this study, FEC-Mel POP and FEC-PBDT POP were synthesized using a condensation reaction in the presence of DMSO. The polymers were created by incorporating a unit called FEC with two different aryl amines, namely Mel and PBDT, without adding any catalysts during their synthesis. Exploring these polymers’ potential uses in CO_2_ uptake and supercapacitors were the main goal of their development. Numerous analytical techniques, including BET (Brunauer–Emmett–Teller), Fourier-transform infrared spectroscopy (FTIR), solid-state nuclear magnetic resonance (ssNMR), powder X-ray diffraction (XRD), and scanning electron microscopy (SEM), were used to validate the synthesis, morphology, and porosity of the FEC-Mel and FEC-PBDT POPs. A capacitance of 82 F g^−1^ was discovered on the FEC-PBDT POP electrode. This result shows that it is feasible to use them in supercapacitors. The FEC-Mel POP sample also showed significant CO_2_ capture capabilities of 1.34 and 1.57 mmol g^−1^ at 298 and 273 K. Overall, the findings point to the potential of the FEC-POPs produced in this work for use in gas adsorption and energy devices.

## 2. Results and Discussion

### 2.1. Synthesis and Molecular Characterization of FEC-CHO, PBDT, and FEC-POPs

**Figure 1:**
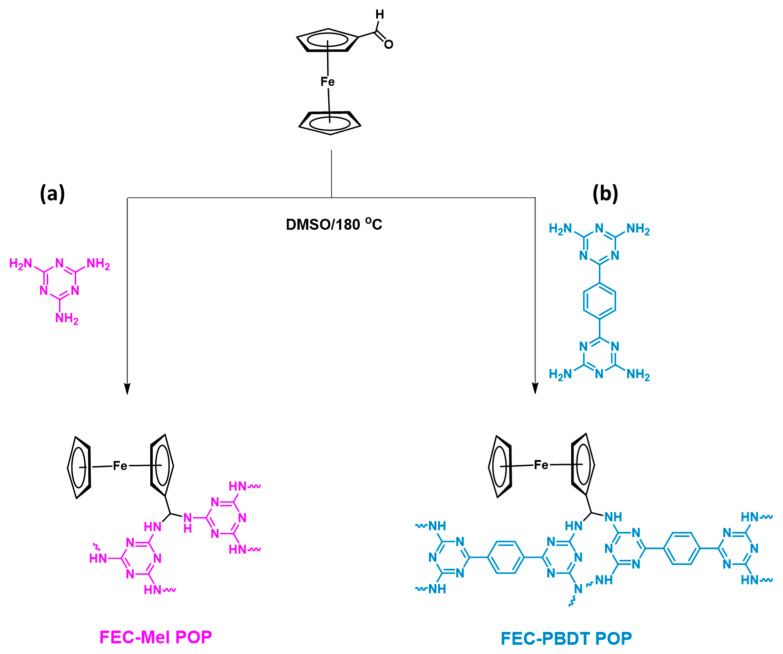
The condensation reaction is used to synthesize (**a**) FEC-Mel and (**b**) FEC-PBDT POPs.

Two porous organic polymers (POPs) incorporating a ferrocene moiety were synthesized using a polycondensation reaction. The building unit, FEC-CHO, was reacted with Mel and PBDT in DMSO at 180 °C to yield FEC-Mel and FEC-PBDT POPs, respectively (see Figure 1a,b). To obtain the FEC-CHO compound, FEC was reacted with POCl_3_ in DMF as a solvent for 16 h, resulting in crimson crystals (Scheme S1a). The FTIR spectrum of FEC-CHO exhibited peaks at 1243 and 1034 cm^−1^, corresponding to the presence of cyclopentadiene rings [Figure S1]. The ^1^H NMR spectrum of FEC-CHO displayed signals at 4.80, 4.67, 4.28, and 9.90 ppm, which were attributed to the cyclopentadiene rings and the aldehyde unit’s carbonyl group, respectively [Figure S2]. The ^13^C NMR analysis also identified signals at 194.32 ppm for the C=O group and at 69.77 and 73.53 ppm for the carbons in the FEC moiety [Figure S3]. For the synthesis of the PBDT monomer, GD-2CN was refluxed with BZ-2CN and KOH in DMF at 130 °C, resulting in a white powder (Scheme S1b). The FTIR spectrum exhibited peaks at 3301 and 3125 cm^−1^ due to the NH_2_ group in PBDT [Figure S4]. The proton signals at 8.35 ppm and 6.90 ppm in the ^1^H NMR spectrum [Figure S5a] corresponded to the aromatic protons and the amino group in the PBDT structure, respectively. Moreover, the ^13^C NMR analysis of PBDT [Figure S5b] revealed signals at 170.91, 168.33, 140.24, and 128.20 ppm, indicating the presence of the carbonyl group, triazine unit, and aromatic ring, respectively. The successful synthesis of the FEC-CHO and PBDT monomers was confirmed through FTIR, ^1^H NMR, and ^13^C NMR analyses. The produced FEC-POPs exhibited remarkable chemical stability and were insoluble in commonly used organic solvents such as MeOH, DCM, acetone, DMF, and EtOH, indicating a high degree of polymerization.

**Figure 2:**
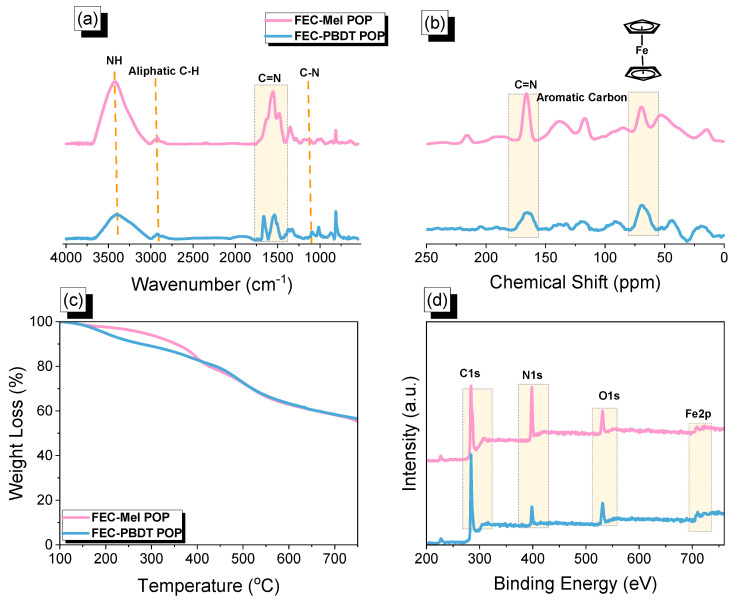
The chemical structure and thermal stability of FEC-Mel and FEC-PBDT POPs obtained by using (**a**) FTIR, (**b**) ssNMR, (**c**) TGA, and (**d**) XPS analyses.

The structures of the obtained FEC POPs (FEC-Mel and FEC-PBDT POPs) were confirmed through solid-state ^13^C NMR spectra and FT-IR measurements. The FT-IR spectra of FEC POPs displayed absorption peaks at 1023 cm^−1^, which corresponded to the C=C stretching of the ferrocene unit. Notably, absorptions at around 1082, 1554, and 3418 cm^−1^ were attributed to C-N, C=N and NH units, respectively. Moreover, distinct vibration bands for the aliphatic C-H group originating from the ferrocene unit were observed at 2927 cm^−1^ (Figure 2a). In the ^13^C NMR profiles of FEC-Mel and FEC-PBDT POPs, signals between 116 and 146 ppm were assigned to carbon atoms in the aromatic rings. Additionally, a signal at 165 ppm indicated the presence of the C=N group in FEC-POPs. Signals ranging from 63 to 76 ppm were assigned to FEC moieties (Figure 2b). Thermogravimetric analysis (TGA) measurements conducted under a nitrogen environment were used to evaluate the thermal characteristics of FEC-POPs (Figure 2c). The TGA profiles of FEC-Mel and FEC-PBDT POPs demonstrated their chemical stability, with decomposition temperatures of 281 and 198 °C, respectively, at 5 wt%, and 353 and 278 °C, respectively, at 10 wt%. Furthermore, the residual weights of FEC-Mel and FEC-PBDT POPs at 800 °C were 52% and 55%, respectively. These results confirmed the high thermal stability of the FEC-POPs. The elemental compositions of the FEC-POPs were determined through X-ray photoelectron spectroscopy (XPS) (Figure 2d). The XPS spectra exhibited signals at 284, 400, and 532 eV, corresponding to the presence of C, N, and O atoms in the structures of the FEC-POPs. Additionally, the peak of the Fe element from the FEC unit appeared at a binding energy of 710 eV [61], validating the successful incorporation of the ferrocene unit into the FEC-POPs networks. Figure S6 illustrates the thermal stability analysis of the FEC-Mel POP sample, conducted via FTIR analysis, across a temperature range from 25 to 200 °C. The results demonstrated that all absorption peaks corresponding to NH, aliphatic C-H, and C=N functionalities remained unchanged. This finding strongly suggests that the FEC-Mel POP sample exhibited excellent thermal stability, even at elevated temperatures.

**Figure 3:**
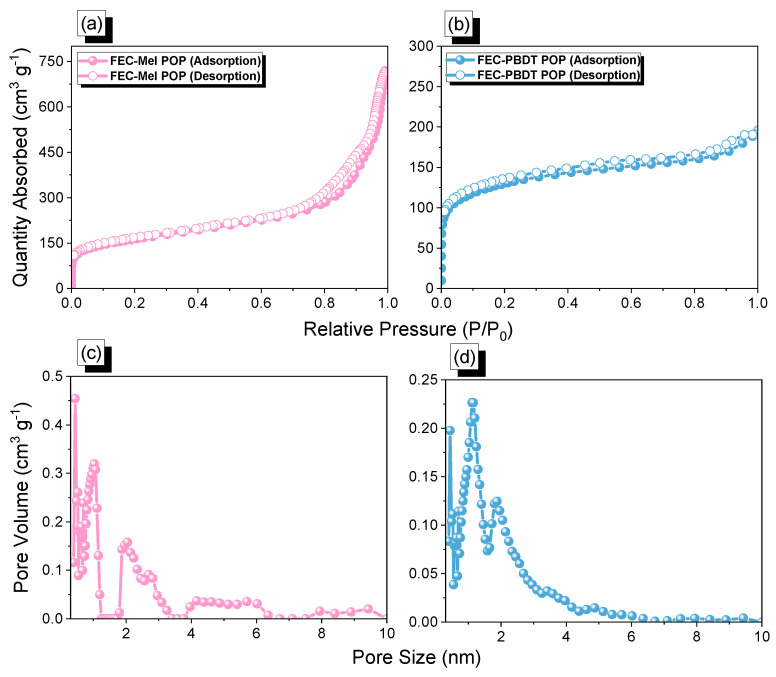
(**a**) N_2_ adsorption/desorption isotherm analyses and (**b**) pore size distributions of FEC-Mel (**a**,**c**) and (**b**,**d**) FEC-PBDT POPs.

Additionally, we conducted further research to evaluate the porosity and surface area of our FEC-POPs materials. Nitrogen sorption measurements, including adsorption and desorption isotherms, were performed to check the porous characteristics of FEC-Mel and FEC-PBDT POPs. The BET isotherms and pore size analysis of FEC-POPs at 77 K are presented in Figure 3. The N_2_ adsorption and desorption curves of both FEC POPs exhibited a type IV isotherm, indicating the presence of micropores and mesopores. Remarkably, FEC-Mel and FEC-PBDT POPs displayed large S_BET_ of 556 and 428 m^2^ g^−1^, respectively (Figure 3a,b). Based on the NL-DFT theory, the pore diameters of FEC-POPs were determined to be in the ranges of 0.41–5.85 nm for FEC-Mel and 0.45–1.85 nm for FEC-PBDT POPs (Figure 3c,d).

**Figure 4:**
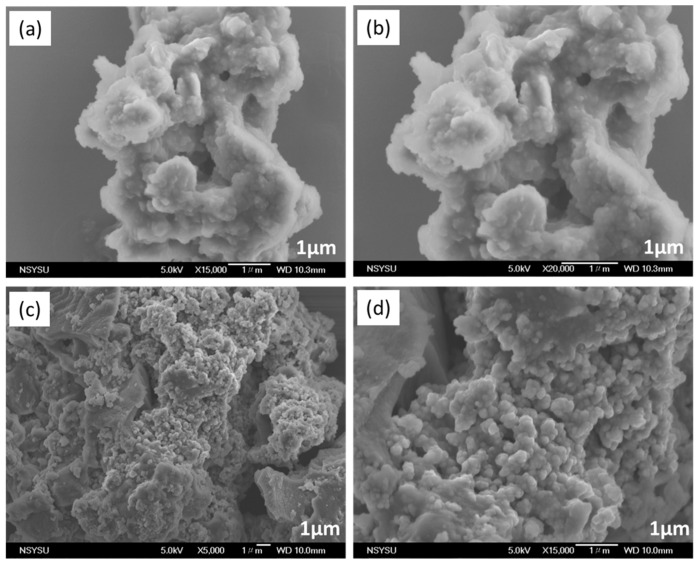
SEM images of FEC-Mel POP (**a**,**b**) and (**c,d**) FEC-PBDT POP.

SEM was employed to investigate the shape morphologies of FEC-POPs. The SEM images revealed irregular spherical particles for both FEC-Mel and FEC-PBDT POPs (Figure 4a–d).

**Figure 5:**
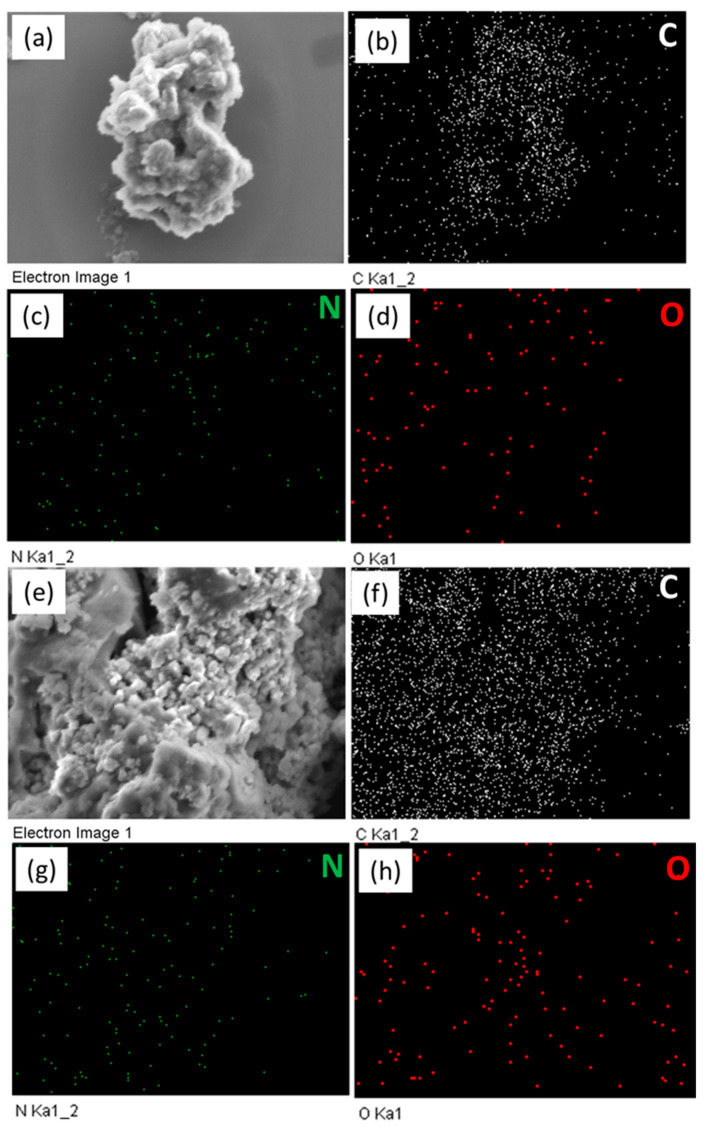
SEM images and their corresponding elemental mapping of FEC-Mel (**a**–**d**) and FEC-PBDT POPs (**e**–**h**). The scale bar in all images is 6 µm.

The SEM-EDS mapping confirmed the presence of carbon (C), nitrogen (N), and oxygen (O) atoms in FEC POPs (Figure 5a–h). Overall, the FEC-Mel and FEC-PBDT POPs exhibited N-heteroatom structures, high surface areas, meso- and microporous characteristics, and significant pore volumes. These findings suggest that these materials hold promise as potential candidates for applications in energy storage and gas capture [63]. XRD analysis (Figure S7) indicated the presence of semi-crystalline peaks and broad diffraction peaks in the XRD profiles, suggesting the structural characteristics of the materials [59].

### 2.2. CO_2_ Uptake of FEC-Mel and FEC-PBDT POPs

**Figure 6:**
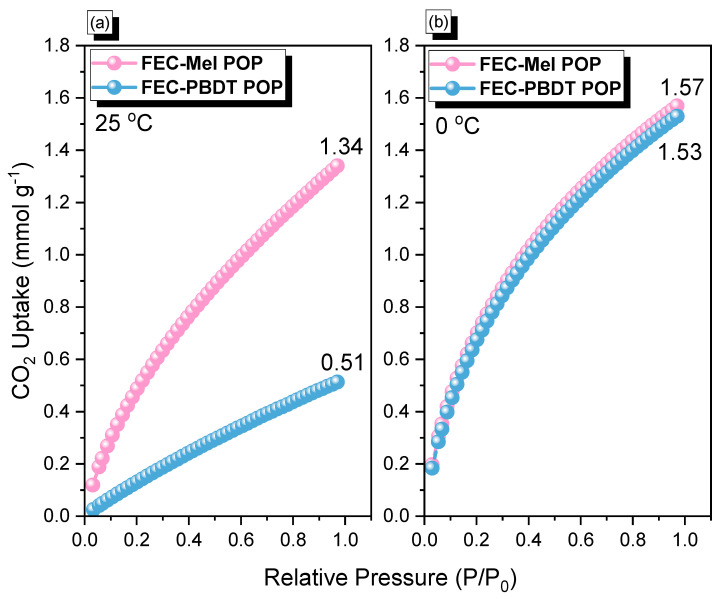
CO_2_ uptake analyses by FEC-Mel and FEC-PBDT POPs at 298 (**a**) and 273 K (**b**).

To evaluate the CO_2_ uptake capabilities of FEC-Mel and FEC-PBDT POPs, CO_2_ isotherm measurements were conducted at temperatures of 298 K and 273 K, respectively. At 298 K, FEC-Mel and FEC-PBDT POPs displayed a CO_2_ capacity of 1.34 and 0. 51 mmol g^−1^, respectively. At 273 K, the CO_2_ uptake capacities increased to 1.57 mmol g^−1^ for FEC-Mel POP and 1.53 mmol g^−1^ for FEC-PBDT POP (Figure 6). The superior CO_2_ uptake performance of FEC-Mel POP can be explained by its high S_BET_ surface area and total pore volume.

### 2.3. Electrochemical Analysis of FEC-POPs

**Figure 7:**
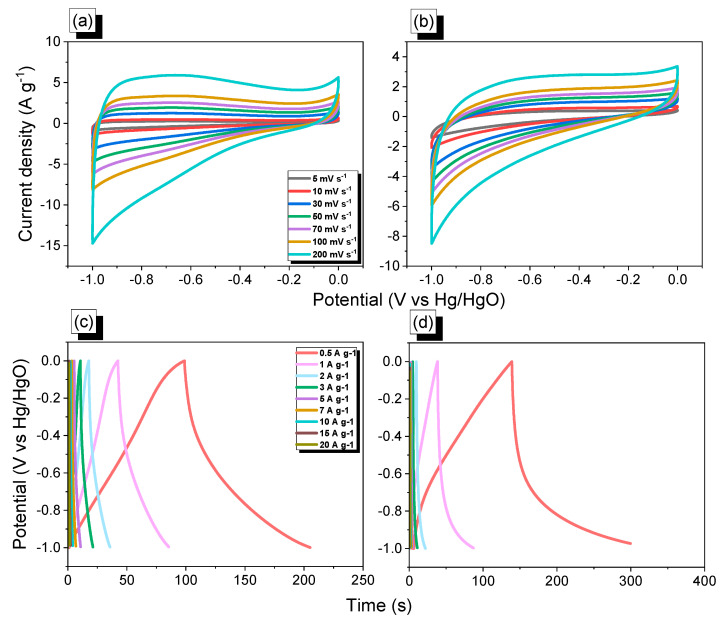
Electrochemical analyses were obtained by using CV (**a**,**b**) and GCD (**c**,**d**) of FEC-Mel (**a**,**c**) and (**b**,**d**) FEC-PBDT POPs.

The electrochemical performance of FEC-Mel and FEC-PBDT POPs warrants investigation owing to their considerable BET surface areas and incorporation of triazine moieties, as illustrated in Figure 1. In this study, we examined the cyclic voltammetry (CV) profiles of FEC-Mel and FEC-PBDT POPs at different scan rates ranging from 5 to 200 mV s^−1^. Additionally, the potential window spanning from −1 to 0 V versus Hg/HgO was explored for both materials. These measurements were conducted using a three-electrode system with 6 M KOH serving as the electrolyte. The obtained results are presented in Figure 7. The CV curves depicted in Figure 7a,b exhibit rectangular shapes without any redox peak for both FEC-Mel and FEC-PBDT POPs, indicating that the electrochemical properties of these materials primarily resemble those of electrical double-layer capacitors (EDLCs). Moreover, our findings demonstrate that the CV behavior of the FEC-Mel and FEC-PBDT POPs samples remains stable and reversible without any distortion across a scanning rate range of 5 to 200 mV s^−1^. This data signifies that these two FEC-POPs materials possess favorable electron transfer rates and ion exchange capabilities. In addition, the capacitance performance of both FEC-Mel and FEC-PBDT POPs samples was assessed using galvanostatic charge–discharge (GCD) measurements at different current densities (ranging from 0.5 to 20 A g^−1^) and all CGD profiles are approximately in an isosceles triangle shape. The results, depicted in Figure 7c,d, clearly demonstrate that the FEC-PBDT POP sample exhibits higher specific capacitance values compared to the FEC-Mel POP sample.

**Figure 8:**
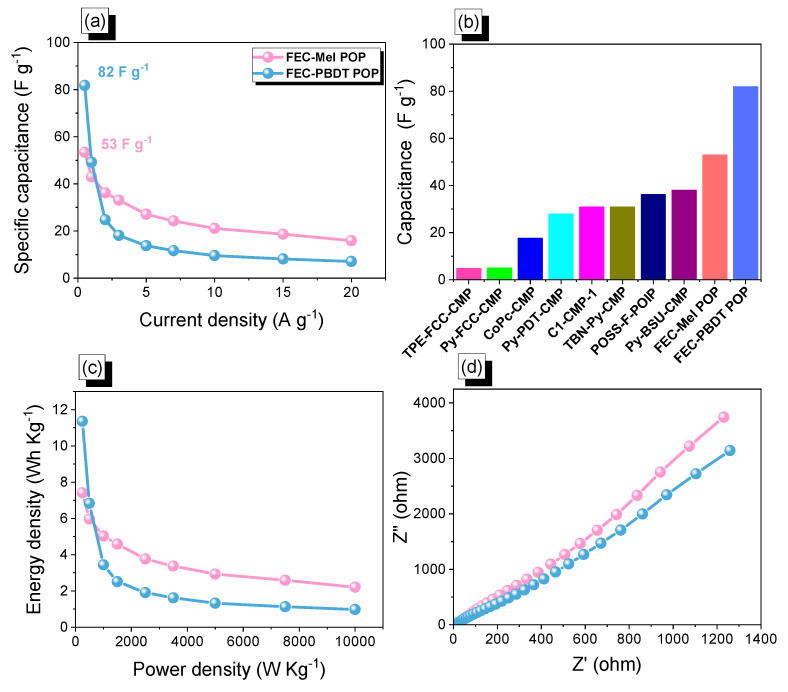
(**a**) Capacitance and (**b**) electrochemical performance of FEC-POPs in comparison to other organic POPs electrodes. (**c**) Ragone analysis and (**d**) electrochemical impedance spectroscopy (EIS) profiles of FEC-Mel and FEC-PBDT POPs.

In Figure 8a, the specific capacitance values for the FEC-Mel POP sample were observed to be 54, 43, 36, 33, 27, 24, 21, 19, and 16 F g^−1^ at a current density of 0.5, 1, 2, 3, 5, 7, 10, 15, and 20 A g^−1^, respectively. Similarly, for the FEC-PBDT POP, the specific capacitance values were recorded as 82, 49, 25, 18, 14, 12, 10, 8, and 7 F g^−1^ at the corresponding discharge rates of 0.5, 1, 2, 3, 5, 7, 10, 15, and 20 A g^−1^. Figure 8b and Table 1 demonstrate the excellent supercapacitor performance of the FEC-PBDT POP compared to other POPs precursors [21,64,65,66,67,68,69]. This superiority can be attributed to two key factors: its high surface area and the increased proportion of nitrogen atoms in the PBDT units. The higher surface area allows for more efficient ion adsorption and desorption, leading to enhanced capacitance. Moreover, the higher content of nitrogen atoms in the PBDT units contributes to improved electrochemical performance, as nitrogen functionalities can facilitate pseudocapacitive behavior and enhance the overall capacitance of the material [14,70,71,72,73,74]. Overall, these characteristics make the FEC-PBDT POP a highly promising candidate for supercapacitor applications.

The Ragone plot shown in Figure 8c compares the energy density of the electrode materials FEC-Mel and FEC-PBDT POPs. The FEC-PBDT material exhibits an energy density of 7.42 Wh kg^−1^, while the FEC-PBDT POPs material has a higher energy density of 11.36 Wh kg^−1^. These values correspond to a power density of 250 W kg^−1^. Notably, both FEC-Mel and FEC-PBDT POPs materials outperform other N-doped porous carbon materials, with an energy density of 7.11 Wh kg^−1^ [75], and a porous graphene carbon material, with an energy density of 2.4 Wh kg^−1^ [76]. This highlights the superior performance of FEC-Mel and FEC-PBDT POPs as highly efficient N-doped porous carbon materials. To comprehend the ion diffusion process and electrical resistance of the electrodes, electrochemical impedance spectroscopy (EIS) was employed. By analyzing Figure 8d, depicting the Nyquist plot, we can assess the resistances exhibited by FEC-Mel POP and FEC-PBDT POP electrodes. Our primary focus was investigating the ohmic resistances of these electrodes, which were determined as 18.50 and 4.20, respectively. In EIS measurements, ohmic resistance pertains to the resistance encountered by the electric current as it flows between the bulk electrolyte and the electrode–electrolyte interface. This resistance comprises several components, such as electrolyte resistance, resistance at the electrode–electrolyte interface, and any other resistances present within the system. In Figure S8, the chemical structure stability of both the FEC-Mel POP and FEC-PBDT POP samples was examined through FTIR analysis after undergoing electrochemical measurements. The results revealed that all absorption peaks associated with NH, aliphatic C-H, and C=N functionalities remained unaltered. This outcome strongly indicates that the FEC-Mel POP sample displayed outstanding chemical stability, even after the electrochemical experiment.

## 3. Materials and Methods

### 3.1. Materials

Ferrocene (FEC), 1,4-dicyanobenzene (BZ-2CN), dimethyl sulfoxide (DMSO), potassium hydroxide (KOH), chloroform, dimethyl formamide (DMF), phosphoryl chloride (POCl_3_), sodium acetate, hexane, acetone, NaOH, 2-cyanoguanidine (GD-2CN), tetrahydrofuran (THF), ethanol (EtOH), and methanol (MeOH) were obtained via various trade resources, such as Sigma-Aldrich (Darmstadt, Germany), and Alfa Aesar (Lancashire, UK).

### 3.2. Synthesis of Ferrocenecarboxaldehyde (FEC-CHO)

POCl_3_ (37 mL, 0.4 mol) was then progressively added after DMF (75 mL, 0.98 mol) had been chilled in an ice bath. A 15 min break was followed by the addition of 100 mL of CHCl_3_ to thin the liquid. FEC (25 g, 0.14 mol) was then included in the mixture. The resultant mixture was agitated at 60 °C (for 16 h) and had a dark amber appearance. Upon completion of the reaction, the mixture was allowed to cool down. Ice water (500 mL) was then added gradually, followed by the slow addition of 35 g of NaOH and 107 g of sodium acetate. The product was extracted using chloroform (500 mL) and subsequently washed three times with water. To purify the product, it was passed through a silica column, and any remaining impurities were removed by elution with a hexane/acetone mixture (ratio of 5/1). Finally, crimson crystals of the desired product were obtained (17 g, yield: 61%, Scheme S1a). FTIR: 1243, 1034 cm^−1^ (Figure S1). ^1^H NMR (Figure S2): 9.90 (1H), 4.80 (2H), 4.67 (2H), 4.28 (5H). ^13^C NMR (Figure S3): 194.32, 69.77, 73.53.

### 3.3. Synthesis of 6,6′-(1,4-Phenylene)bis(1,3,5-Triazine-2,4-Diamine) (PBDT)

A reaction was carried out using the following procedure: In a flask, BZ-2CN (0.386 g, 2.3 mmol) was added into DMF (10 mL). Separately, a mixture of KOH (0.281 g, 5 mmol) and GD-2CN (1.012 g, 12 mmol) was added into DMF (40 mL). The KOH/GD-2CN solution was then added to the BZ-2CN solution. About 20 h were spent stirring and refluxing the resultant mixture at 130 °C under a N_2_. Once the reaction was complete, the product was subjected to thorough washing with EtOH and MeOH to obtain PBDT (Scheme S1b). FTIR (Figure S4): 3301, 3125, and 1550. ^1^H NMR (Figure S5a, δ, ppm) 8.35, 6.90. ^13^C NMR (Figure S5b, δ, ppm) 170.91, 168.33, 140.24, 128.20.

### 3.4. Synthesis of FEC-Mel POP

Mel (0.5 g, 3.96 mmol) and FEC-CHO (1.5 g, 7.01 mmol) in DMSO (20 mL) were produced and charged into a Schlenk flask. Three cycles of freezing and thawing were performed on the flask. The reaction flask was then heated to 180 °C and held at this temperature while stirring for 3 days. Following the reaction, the flask was allowed to cool to room temperature. After filtering, the finished product was washed with acetone, MeOH, and THF. The resulting black powder is known as FEC-Mel POP (Figure 1a). To prepare FEC-PBDT POP, FEC-CHO (0.87 g, 4.06 mmol) and PBDT (0.5 g, 1.69 mmol) were mixed in 20 mL of DMSO at 180 °C for 3 days. After the reaction period, the flask was allowed to cool down. The resulting product was filtered and washed sequentially with THF, MeOH, and acetone. The obtained solid material, which appeared black, was further dried at 100 °C. As a result, FEC-PBDT POP was obtained as a black powder (Figure 1b).

## 4. Conclusions

In summary, we developed two types of porous organic polymers (FEC-POPs) by incorporating the FEC unit with different aryl amines through a polycondensation reaction. The resulting polymers were named FEC-Mel and FEC-PBDT POPs. Both the FEC-Mel and FEC-PBDT POPs exhibited remarkable thermal stability, with a *T_d_*_10_ value of up to 353 °C and a char yield of approximately 54.55 wt% at 800 °C, as determined using thermal gravimetric analysis (TGA). For the potential applications for FEC-POPs, we revealed that the FEC-PBDT POP electrode exhibited an exceptional capacitance of 82 F g^−1^, confirming its suitability for application in supercapacitors. Furthermore, the FEC-Mel POP sample demonstrated impressive CO_2_ capture capacities, measuring 1.34 and 1.57 mmol g^−1^ at 298 and 273 K, respectively. These findings highlight the potential of the synthesized FEC-POPs for utilization in energy storage and gas adsorption devices.

## Figures and Tables

**Table 1:** The capacity values of FEC-Mel and FEC-PBDT POPs were compared with those reported for various three-electrode supercapacitor materials.

Material	Surface Area (m^2^ g^−1^)	Capacitance	References
FEC-Mel POP	556	53 F g^−1^/0.5 A g^−1^	This work
FEC-PBDT POP	428	82 F g^−1^/0.5 A g^−1^	This work
TBN-Py-CMP	473	31 F g^−1^/0.5 A g^−1^	[67]
CoPc-CMP	-	13.7 F g^−1^/1.0 A g^–1^	[65]
Py-BSU CMP	42	38 F g^−1^/0.5 A g^−1^	[69]
POSS-F-POIP	452	36.2 F g^−1^/0.5 A g^−1^	[68]
TPE-FFC-CMP	8	4.8 F g^−1^/0.5 A g^−1^	[64]
Py-FFC-CMP	50	5.07 F g^−1^/0.5 A g^−1^	[64]
C1-CMP-1	608	31 F g^−1^ at 20 mV s^𢈒1^	[66]
Py-PDT POP	76	28 F g^−1^/0.5 A g^−1^	[21]

## Data Availability

The data presented in this study are available on request from the corresponding author.

## Supplementary Materials

The supporting information can be downloaded at: https://www.mdpi.com/article/10.3390/ijms241512371/s1.

## References

[1] Bhanja P., Das S.K., Bhunia K., Pradhan D., Hayashi T., Hijikata Y., Irle S., Bhaumik A. (2018). A new porous polymer for highly efficient capacitive energy storage. ACS Sustain. Chem. Eng..

[2] Mohamed M.G., Elewa A.M., Li M.S., Kuo S.W. (2023). Construction and multifunctional of hypercrosslinked porous organic polymers containing ferrocene unit for high-performance iodine adsorption and supercapacitor. J. Taiwan Inst. Chem. Eng..

[3] Ejaz M., Mohamed M.G., Chang W.C., Kuo S.W. (2023). Synthesis and design of hypercrosslinked porous organic frameworks containing tetraphenylpyrazine unit for high-performance supercapacitor. J. Polym. Sci..

[4] Yu K., Pan X., Zhang G., Liao X., Zhou X., Yan M., Xu L., Mai L. (2018). Nanowires in energy storage devices: Structures, synthesis, and applications. Adv. Energy Mater..

[5] Lin Z., Goikolea E., Balducci A., Naoi K., Taberna P.-L., Salanne M., Yushin G., Simon P. (2018). Materials for supercapacitors: When Li-ion battery power is not enough. Mater. Today.

[6] Chen D., Jiang K., Huang T., Shen G. (2020). Recent advances in fiber supercapacitors: Materials, device configurations, and applications. Adv. Mater..

[7] Zhao H., Wang J., Zheng Y., Li J., Han X., He G., Du Y. (2017). Organic thiocarboxylate electrodes for a room-temperature sodium-ion battery delivering an ultrahigh capacity. Angew. Chem..

[8] Eftekhari A., Fang B. (2017). Electrochemical hydrogen storage: Opportunities for fuel storage, batteries, fuel cells, and supercapacitors. Int. J. Hydrogen Energy.

[9] Simon P., Gogotsi Y. (2008). Materials for electrochemical capacitors. Nat. Mater..

[10] Sarangapani S., Tilak B., Chen C.P. (1996). Materials for electrochemical capacitors: Theoretical and experimental constraints. J. Electrochem. Soc..

[11] Kim D., Kang J., Yan B., Seong K.-d., Piao Y. (2020). Ambient temperature synthesis of iron-doped porous nickel pyrophosphate nanoparticles with long-term chemical stability for high-performance oxygen evolution reaction catalysis and supercapacitors. ACS Sustain. Chem. Eng..

[12] Septiani N.L.W., Kaneti Y.V., Fathoni K.B., Wang J., Ide Y., Yuliarto B., Dipojono H.K., Nanjundan A.K., Golberg D., Bando Y. (2020). Self-assembly of nickel phosphate-based nanotubes into two-dimensional crumpled sheet-like architectures for high-performance asymmetric supercapacitors. Nano Energy.

[13] Shi R., Han C., Duan H., Xu L., Zhou D., Li H., Li J., Kang F., Li B., Wang G. (2018). Redox-active organic sodium anthraquinone-2-sulfonate (AQS) anchored on reduced graphene oxide for high-performance supercapacitors. Adv. Energy Mater..

[14] Samy M.M., Mohamed M.G., Kuo S.-W. (2020). Directly synthesized nitrogen-and-oxygen–doped microporous carbons derived from a bio-derived polybenzoxazine exhibiting high-performance supercapacitance and CO_2_ uptake. Eur. Polym. J..

[15] D’Alessandro D.M., Smit B., Long J.R. (2010). Carbon dioxide capture: Prospects for new materials. Angew. Chem. Int. Ed..

[16] Wang Q., Luo J., Zhong Z., Borgna A. (2011). CO_2_ capture by solid adsorbents and their applications: Current status and new trends. Energy Environ. Sci..

[17] Rochelle G.T. (2009). Amine scrubbing for CO_2_ capture. Science.

[18] Rao A.B., Rubin E.S. (2002). A technical, economic, and environmental assessment of amine-based CO_2_ capture technology for power plant greenhouse gas control. Environ. Sci. Technol..

[19] Mukherjee S., Das M., Manna A., Krishna R., Das S. (2019). Dual strategic approach to prepare defluorinated triazole-embedded covalent triazine frameworks with high gas uptake performance. Chem. Mater..

[20] Lv H., Wang W., Li F. (2018). Porous organic polymers with built-in N-heterocyclic carbenes: Selective and efficient heterogeneous catalyst for the reductive N-formylation of amines with CO_2_. Chem. Eur. J..

[21] Mousa A.O., Mohamed M.G., Chuang C.-H., Kuo S.-W. (2023). Carbonized Aminal-Linked Porous Organic Polymers Containing Pyrene and Triazine Units for Gas Uptake and Energy Storage. Polymers.

[22] Cui Y., Du J., Liu Y., Yu Y., Wang S., Pang H., Liang Z., Yu J. (2018). Design and synthesis of a multifunctional porous N-rich polymer containing s-triazine and Tröger’s base for CO_2_ adsorption, catalysis and sensing. Polym. Chem..

[23] McKeown N.B., Budd P.M., Book D. (2007). Microporous polymers as potential hydrogen storage materials. Macromol. Rapid Commun..

[24] Zhu Y., Xu P., Zhang X., Wu D. (2022). Emerging porous organic polymers for biomedical applications. Chem. Soc. Rev..

[25] Das S., Heasman P., Ben T., Qiu S. (2017). Porous organic materials: Strategic design and structure–function correlation. Chem. Rev..

[26] Zhang T., Xing G., Chen W., Chen L. (2020). Porous organic polymers: A promising platform for efficient photocatalysis. Mater. Chem. Front..

[27] Yuan D., Lu W., Zhao D., Zhou H.C. (2011). Highly stable porous polymer networks with exceptionally high gas-uptake capacities. Adv. Mater..

[28] Wang T.-X., Liang H.-P., Anito D.A., Ding X., Han B.-H. (2020). Emerging applications of porous organic polymers in visible-light photocatalysis. J. Mater. Chem. A.

[29] Xu Y., Mao N., Feng S., Zhang C., Wang F., Chen Y., Zeng J., Jiang J.X. (2017). Perylene-Containing Conjugated Microporous Polymers for Photocatalytic Hydrogen Evolution. Macromol. Chem. Phys..

[30] Mohamed M.G., Tsai M.-Y., Wang C.-F., Huang C.-F., Danko M., Dai L., Chen T., Kuo S.-W. (2021). Multifunctional polyhedral oligomeric silsesquioxane (POSS) based hybrid porous materials for CO_2_ uptake and iodine adsorption. Polymers.

[31] Mousa A.O., Lin Z.-I., Chuang C.-H., Chen C.-K., Kuo S.-W., Mohamed M.G. (2023). Rational Design of Bifunctional Microporous Organic Polymers Containing Anthracene and Triphenylamine Units for Energy Storage and Biological Applications. Int. J. Mol. Sci..

[32] Mohamed M.G., Elsayed M.H., Ye Y., Samy M.M., Hassan A.E., Mansoure T.H., Wen Z., Chou H.-H., Chen K.-H., Kuo S.-W. (2023). Construction of Porous Organic/Inorganic Hybrid Polymers Based on Polyhedral Oligomeric Silsesquioxane for Energy Storage and Hydrogen Production from Water. Polymers.

[33] Dawson R., Cooper A.I., Adams D.J. (2013). Chemical functionalization strategies for carbon dioxide capture in microporous organic polymers. Polym. Int..

[34] Gao H., Li Q., Ren S. (2019). Progress on CO_2_ capture by porous organic polymers. Curr. Opin. Green Sustain. Chem..

[35] Chen D., Fu Y., Yu W., Yu G., Pan C. (2018). Versatile Adamantane-based porous polymers with enhanced microporosity for efficient CO_2_ capture and iodine removal. Chem. Eng. J..

[36] Liu C., Jin Y., Yu Z., Gong L., Wang H., Yu B., Zhang W., Jiang J. (2022). Transformation of porous organic cages and covalent organic frameworks with efficient iodine vapor capture performance. J. Am. Chem. Soc..

[37] Abid A., Razzaque S., Hussain I., Tan B. (2021). Eco-Friendly Phosphorus and Nitrogen-Rich Inorganic-Organic Hybrid Hypercross-linked Porous Polymers via a Low-Cost Strategy. Macromolecules.

[38] Mohamed M.G., EL-Mahdy A.F., Kotp M.G., Kuo S.-W. (2022). Advances in porous organic polymers: Syntheses, structures, and diverse applications. Mater. Adv..

[39] Zhang W., Mu Y., He X., Chen P., Zhao S., Huang C., Wang Y., Chen J. (2020). Robust porous polymers bearing phosphine oxide/chalcogenide ligands for volatile iodine capture. Chem. Eng. J..

[40] Zou X., Ren H., Zhu G. (2013). Topology-directed design of porous organic frameworks and their advanced applications. Chem. Commun..

[41] Ben T., Ren H., Ma S., Cao D., Lan J., Jing X., Wang W., Xu J., Deng F., Simmons J.M. (2009). Targeted synthesis of a porous aromatic framework with high stability and exceptionally high surface area. Angew. Chem. Int. Ed..

[42] Ding L., Gao H., Xie F., Li W., Bai H., Li L. (2017). Porosity-enhanced polymers from hyper-cross-linked polymer precursors. Macromolecules.

[43] Sun L., Zou Y., Liang Z., Yu J., Xu R. (2014). A one-pot synthetic strategy via tandem Suzuki–Heck reactions for the construction of luminescent microporous organic polymers. Polym. Chem..

[44] Pan L., Chen Q., Zhu J.-H., Yu J.-G., He Y.-J., Han B.-H. (2015). Hypercrosslinked porous polycarbazoles via one-step oxidative coupling reaction and Friedel-Crafts alkylation. Polym. Chem..

[45] Fang D., Li X., Zou M., Guo X., Zhang A. (2019). Carbazole-functionalized hyper-cross-linked polymers for CO2 uptake based on Friedel-Crafts polymerization on 9-phenylcarbazole. Beilstein J. Org. Chem..

[46] Kuo S.W. (2023). Construction Archimedean tiling patterns based on soft materials from block copolymers and covalent organic frameworks. Giant.

[47] Liu M., Guo L., Jin S., Tan B. (2019). Covalent triazine frameworks: Synthesis and applications. J. Mater. Chem. A.

[48] Chung W.T., Mekhemer I.M.A., Mohamed M.G., Elewa A.M., EL-Mahdy A.F.M., Chou H.H., Kuo S.W., Wu K.C.W. (2023). Recent advances in metal/covalent organic frameworks based materials: Their synthesis, structure design and potential applications for hydrogen production. Coord. Chem. Rev..

[49] Samy M.M., Mohamed M.G., Kuo S.-W. (2023). Conjugated Microporous Polymers Based on Ferrocene Units as Highly Efficient Electrodes for Energy Storage. Polymers.

[50] Meng Z., Sato K., Sukegawa T., Oyaizu K., Ho C.-L., Xiang J., Feng Y.-H., Lo Y.H., Nishide H., Wong W.-Y. (2016). Metallopolyyne polymers with ferrocenyl pendant ligands as cathode-active materials for organic battery application. J. Organomet. Chem..

[51] Cong G., Zhou Y., Li Z., Lu Y.-C. (2017). A highly concentrated catholyte enabled by a low-melting-point ferrocene derivative. ACS Energy Lett..

[52] Zhang C., Qian Y., Ding Y., Zhang L., Guo X., Zhao Y., Yu G. (2019). Biredox eutectic electrolytes derived from organic redox-active molecules: High-energy storage systems. Angew. Chem. Int. Ed..

[53] Elsler B., Schollmeyer D., Dyballa K.M., Franke R., Waldvogel S.R. (2014). Metal-and Reagent-Free Highly Selective Anodic Cross-Coupling Reaction of Phenols. Angew. Chem. Int. Ed..

[54] Mandai H., Fujii K., Yasuhara H., Abe K., Mitsudo K., Korenaga T., Suga S. (2016). Enantioselective acyl transfer catalysis by a combination of common catalytic motifs and electrostatic interactions. Nat. Commun..

[55] Choi T.-L., Lee K.-H., Joo W.-J., Lee S., Lee T.-W., Chae M.Y. (2007). Synthesis and nonvolatile memory behavior of redox-active conjugated polymer-containing ferrocene. J. Am. Chem. Soc..

[56] Dong Q., Meng Z., Ho C.-L., Guo H., Yang W., Manners I., Xu L., Wong W.-Y. (2018). A molecular approach to magnetic metallic nanostructures from metallopolymer precursors. Chem. Soc. Rev..

[57] Tan J., Li H., Hu X., Abdullah R., Xie S., Zhang L., Zhao M., Luo Q., Li Y., Sun Z. (2019). Size-tunable assemblies based on ferrocene-containing DNA polymers for spatially uniform penetration. Chem.

[58] Xiang J., Li X., Ma Y., Zhao Q., Ho C.-L., Wong W.-Y. (2018). Efficient flash memory devices based on non-conjugated ferrocene-containing copolymers. J. Mater. Chem. C.

[59] Wang Y., Tao J., Xiong S., Lu P., Tang J., He J., Javaid M.U., Pan C., Yu G. (2020). Ferrocene-based porous organic polymers for high-affinity iodine capture. Chem. Eng. J..

[60] Ma L., Liu Y., Liu Y., Jiang S., Li P., Hao Y., Shao P., Yin A., Feng X., Wang B. (2019). Ferrocene-linkage-facilitated charge separation in conjugated microporous polymers. Angew. Chem. Int. Ed..

[61] Tan Z., Su H., Guo Y., Liu H., Liao B., Amin A.M., Liu Q. (2020). Ferrocene-based conjugated microporous polymers derived from yamamoto coupling for gas storage and dye removal. Polymers.

[62] Samy M.M., Sharma S.U., Mohamed M.G., Mohammed A.A., Chaganti S.V., Lee J.-T., Kuo S.-W. (2022). Conjugated microporous polymers containing ferrocene units for high carbon dioxide uptake and energy storage. Mater. Chem. Phys..

[63] Dutta S., Bhaumik A., Wu K.C.-W. (2014). Hierarchically porous carbon derived from polymers and biomass: Effect of interconnected pores on energy applications. Energy Environ. Sci..

[64] Samy M.M., Mohamed M.G., Mansoure T.H., Meng T.S., Khan M.A.R., Liaw C.-C., Kuo S.-W. (2022). Solid state chemical transformations through ring-opening polymerization of ferrocene-based conjugated microporous polymers in host–guest complexes with benzoxazine-linked cyclodextrin. J. Taiwan Inst. Chem. Eng..

[65] Mei L., Cui X., Duan Q., Li Y., Lv X., Wang H.-g. (2020). Metal phthalocyanine-linked conjugated microporous polymer hybridized with carbon nanotubes as a high-performance flexible electrode for supercapacitors. Int. J. Hydrogen Energy.

[66] Lee J.-S.M., Wu T.-H., Alston B.M., Briggs M.E., Hasell T., Hu C.-C., Cooper A.I. (2016). Porosity-engineered carbons for supercapacitive energy storage using conjugated microporous polymer precursors. J. Mater. Chem. A.

[67] Samy M.M., Mohamed M.G., El-Mahdy A.F.M., Mansoure T.H., Wu K.C.-W., Kuo S.-W. (2021). High-performance supercapacitor electrodes prepared from dispersions of tetrabenzonaphthalene-based conjugated microporous polymers and carbon nanotubes. ACS Appl. Mater. Interfaces.

[68] Mohamed M.G., Mansoure T.H., Takashi Y., Samy M.M., Chen T., Kuo S.-W. (2021). Ultrastable porous organic/inorganic polymers based on polyhedral oligomeric silsesquioxane (POSS) hybrids exhibiting high performance for thermal property and energy storage. Microporous Mesoporous Mater..

[69] Mohamed M.G., Chang S.-Y., Ejaz M., Samy M.M., Mousa A.O., Kuo S.-W. (2023). Design and Synthesis of Bisulfone-Linked Two-Dimensional Conjugated Microporous Polymers for CO_2_ adsorption and Energy Storage. Molecules.

[70] Samy M.M., Mohamed M.G., Sharma S.U., Chaganti S.V., Lee J.T., Kuo S.W. (2023). An Ultrastable Tetrabenzonaphthalene-Linked conjugated microporous polymer functioning as a high-performance electrode for supercapacitors. J. Taiwan Inst. Chem. Eng..

[71] Ejaz M., Mohamed M.G., Kuo S.W. (2023). Solid state chemical transformation provides a fully benzoxazine-linked porous organic polymer displaying enhanced CO_2_ capture and supercapacitor performance. Polym. Chem..

[72] Samy M.M., Mohamed M.G., Sharma S.U., Chaganti S.V., Mansoure T.H., Lee J.T., Chen T., Kuo S.W. (2023). Constructing conjugated microporous polymers containing triphenylamine moieties for high-performance capacitive energy storage. Polymer.

[73] Chen J., Lin Y., Liu J., Wu D., Bai X., Chen D., Li H. (2021). Outstanding supercapacitor performance of nitrogen-doped activated carbon derived from shaddock peel. J. Energy Storage.

[74] Liu F., Niu J., Chuan Y., Zhao Y. (2023). Nitrogen and sulfur co-doping carbon in different dimensions as electrode for supercapacitor applications. J. Alloys Compd..

[75] Chen L.F., Zhang X.D., Liang H.W., Kong M., Guan Q.F., Chen P., Wu Z.Y., Yu S.H. (2012). Synthesis of Nitrogen-Doped Porous Carbon Nanofibers as an Efficient Electrode Material for Supercapacitors. ACS Nano.

[76] Lei Z., Christov N., Zhao X.S. (2011). Intercalation of mesoporous carbon spheres between reduced graphene oxide sheets for preparing high-rate supercapacitor electrodes. Energy Environ. Sci..

