# Supplementary material for: Strategic Design and Synthesis of Ferrocene Linked Porous Organic Frameworks toward Tunable CO2 Capture and Energy Storage
Source: Int J Mol Sci. 2023 Aug 2;24(15):12371. doi: 10.3390/ijms241512371 (PMC10419241; doi:10.3390/ijms241512371)
Supplement: Supplementary file 1 [file ijms-24-12371-s001.zip › ijms-2494564-supplementary.pdf]

Supporting Information

Strategic Design and Synthesis of Ferrocene Linked Porous Organic Frameworks toward Tunable CO_2 Capture and Energy Storage

Aya Osama Mousa [1,2], Cheng-Hsin Chuang [2], Shiao-Wei Kuo [1,3,*] and Mohamed Gamal Mohamed [1,4,*]

[1] Department of Materials and Optoelectronic Science, Center of Crystal Research, National Sun Yat-sen University, Kaohsiung 804, Taiwan; aya623514@gmail.com
[2] Institute of Medical Science and Technology, National Sun Yat-sen University, Kaohsiung 804201, Taiwan; chchuang@imst.nsysu.edu.tw
[3] Department of Medicinal and Applied Chemistry, Kaohsiung Medical University, Kaohsiung 807, Taiwan
[4] Chemistry Department, Faculty of Science, Assiut University, Assiut 71516, Egypt
* Correspondence: kuosw@faculty.nsysu.edu.tw (S.-W.K.); mgamal.eldin34@gmail.com (M.G.M.)

Characterization

FTIR spectra were collected on a Bruker Tensor 27 FTIR spectrophotometer with a resolution of 4 cm^{-1} by using KBr disk method. ^{13}C nuclear magnetic resonance (NMR) spectra were examined by using an INOVA 500 instrument with DMSO as the solvent and TMS as the external standard. Chemical shifts are reported in parts per million (ppm). The thermal stabilities of the samples were performed by using a TG Q-50 thermogravimetric analyzer under a N_2 atmosphere; the cured sample (ca. 5 mg) was put in a Pt cell with heating rate of 20 °C min^{-1} from 100 to 800 °C under a N_2 flow rate of 60 mL min^{-1}. Wide-angle X-ray diffraction (WAXD) patterns were measured by the wiggler beamline BL17A1 of the National Synchrotron Radiation Research Center (NSRRC), Taiwan. A triangular bent Si (111) single crystal was used to get a monochromated beam having a wavelength (λ) of 1.33 Å. The morphologies of the polymer samples were examined by Field emission scanning electron microscopy (FE-SEM; JEOL JSM7610F) and also by transmission

electron microscope (TEM) using a JEOL-2100 instrument at an accelerating voltage of 200 kV. BET surface area and porosimetry measurements of samples (ca. 40–100 mg) were measured using BEL Master™/BEL sim™ (v. 3.0.0). N_2 adsorption and desorption isotherms were generated through incremental exposure to ultrahigh-purity N_2 (up to ca. 1 atm) in a liquid N_2 (77 K) bath. Surface parameters were calculated using BET adsorption models in the instrument's software. The pore size of the prepared samples was determined by using nonlocal density functional theory (NLDFT).

Electrochemical Analysis

Working Electrode Cleaning: Prior to using, the glassy carbon electrode (GCE) was polished several times with 0.05-μm alumina powder, washed with EtOH after each polishing step, cleaned through sonication (5 min) in a water bath, washed with EtOH, and then dried in the oven at 50 °C.

Electrochemical Characterization: The electrochemical experiments were performed in a three-electrode cell using an Autolab potentiostat (PGSTAT204) and 1 M KOH as the aqueous electrolyte. The GCE was used as the working electrode (diameter: 5.61 mm; 0.2475 cm^2); a Pt wire was used as the counter electrode; Hg/HgO (RE-1B, BAS) was the reference electrode. All reported potentials refer to the Hg/HgO potential. A slurry was prepared by dispersing FEC-Mel or FEC-PBDT POPs (2 mg), carbon black (2 mg), and Nafion (10 wt%) in a mixture of (EtOH/

H_2O) (200 μL: 800 μL) and then sonicating for 1 h. A portion of this slurry (10 μL) was pipetted onto the tip of the electrode, which was then dried in air for 30 min prior to use. The electrochemical performance was studied through CV at various sweep rates (5–200 mV s^{-1}) and through the GCD method in the potential range from 0 to -1.00 V (vs. Hg/HgO) at various current densities (0.5–20 A g^{-1}) in 1 M KOH as the aqueous electrolyte solution.

The specific capacitance was calculated from the GCD data using the equation.

$$C_s = (I\Delta t)/(m\Delta V)$$

Where C_s (F g^{-1}) is the specific capacitance of the supercapacitor, I (A) is the discharge current, ΔV (V) is the potential window, Δt (s) is the discharge time, and m (g) is the mass of the NPC on the electrode. The energy density (E, W h kg^{-1}) and power density (P, W kg^{-1}) were calculated using the equations.

$$E = 1000C(\Delta V)^2/(2 \times 3600)$$

$$P = E/(t/3600)$$

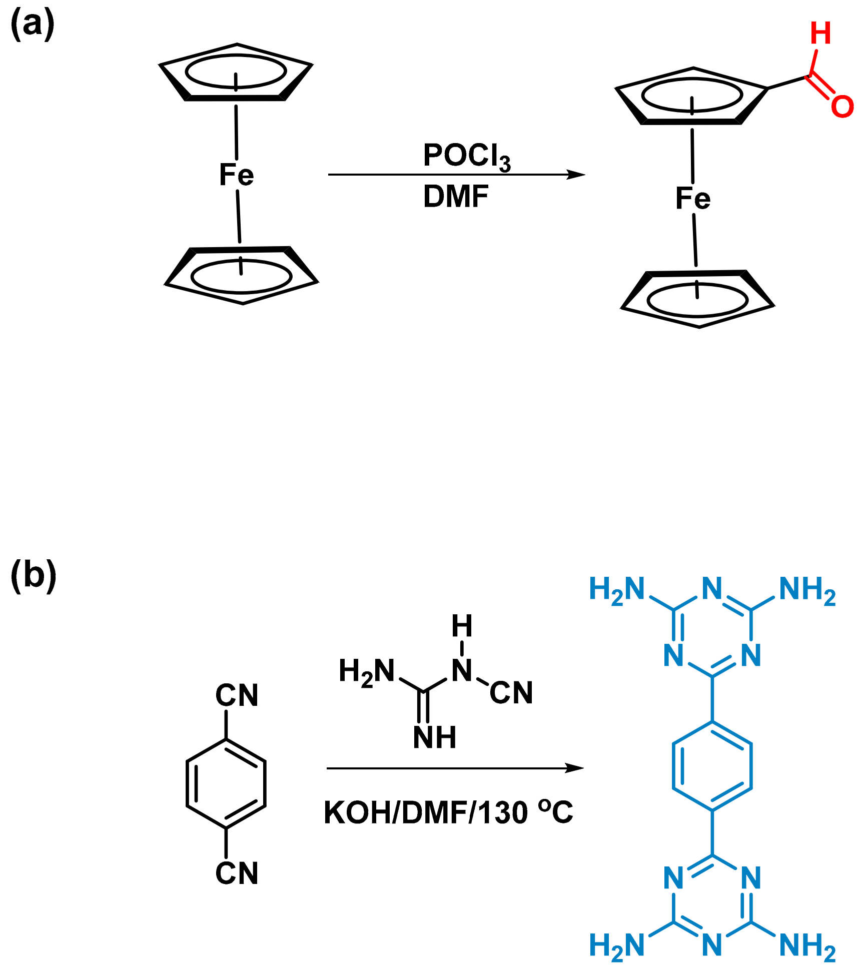

Scheme S1. Synthesis of (a) FEC-CHO and (b) PBDT.

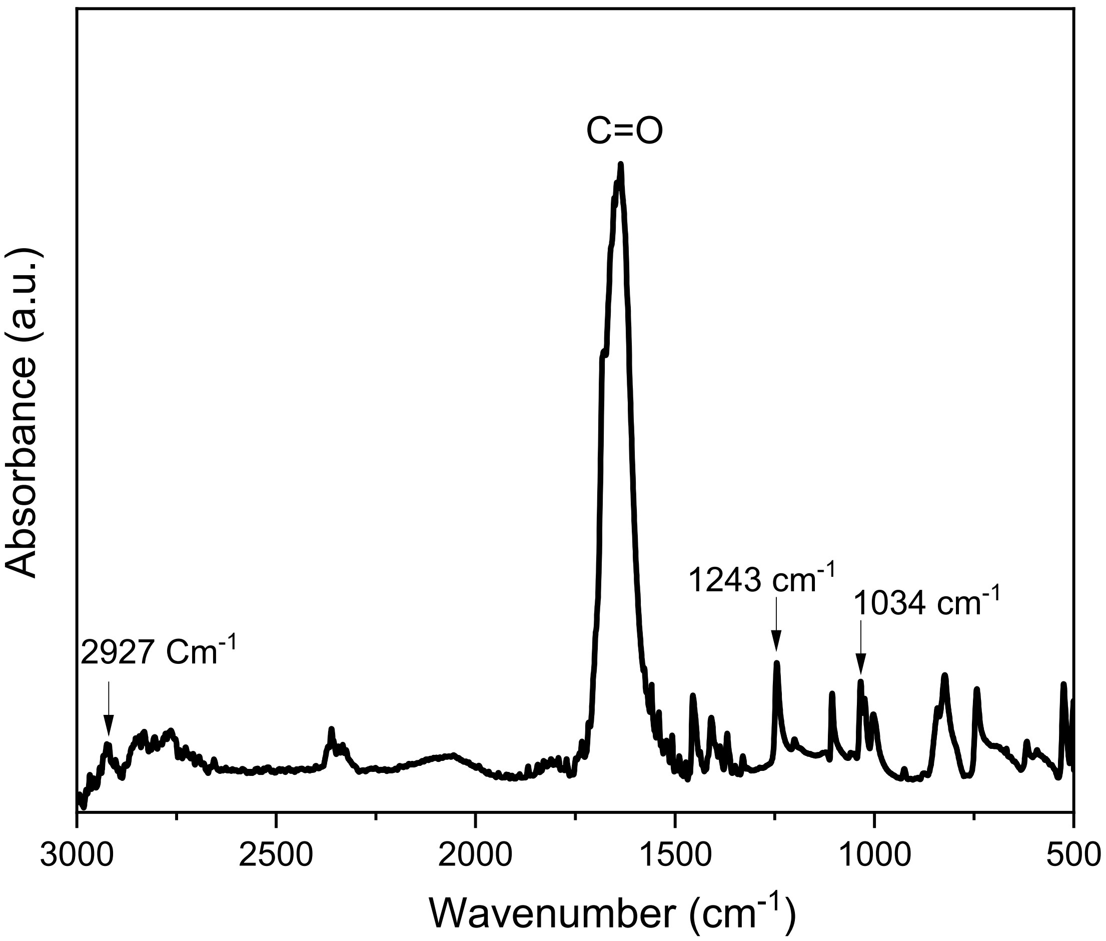

Figure S1. FTIR spectrum of FEC-CHO.

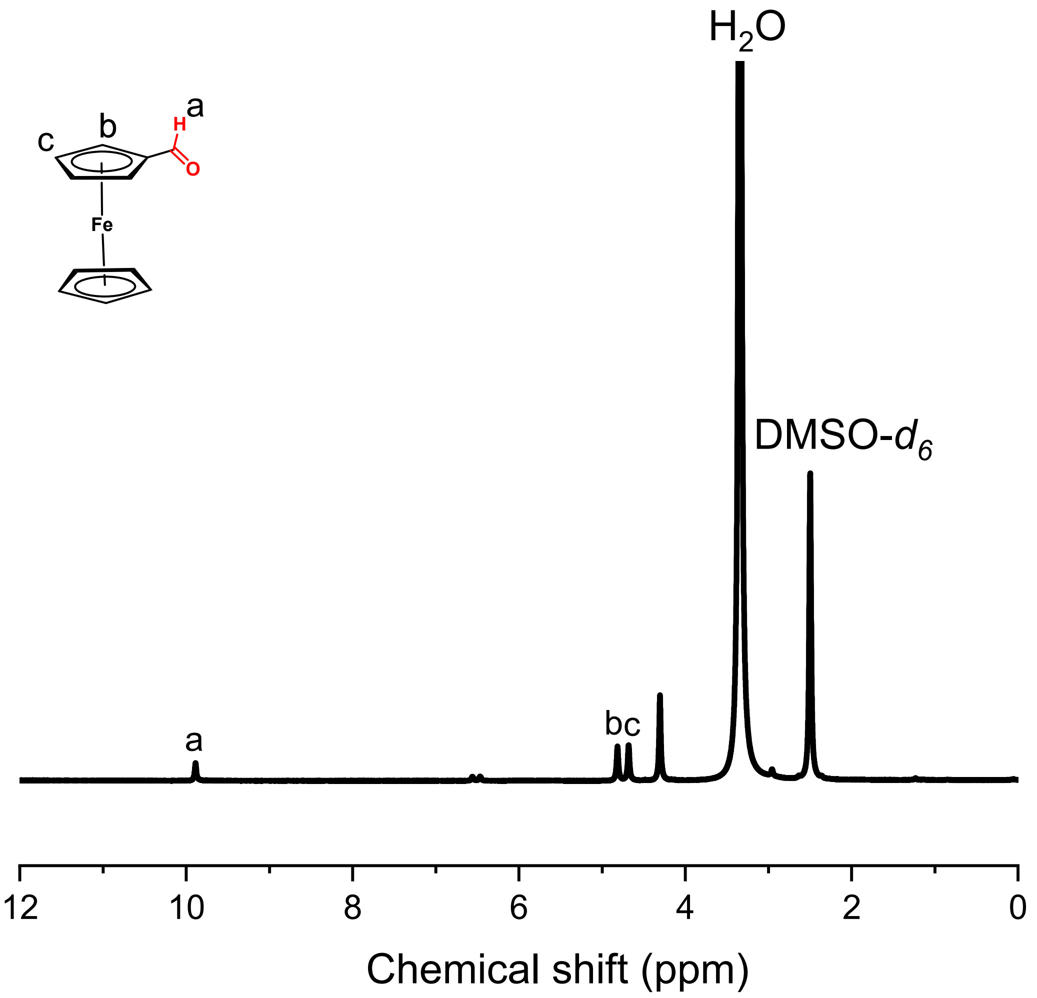

Figure S2. ^{1}H-NMR spectrum of FEC-CHO.

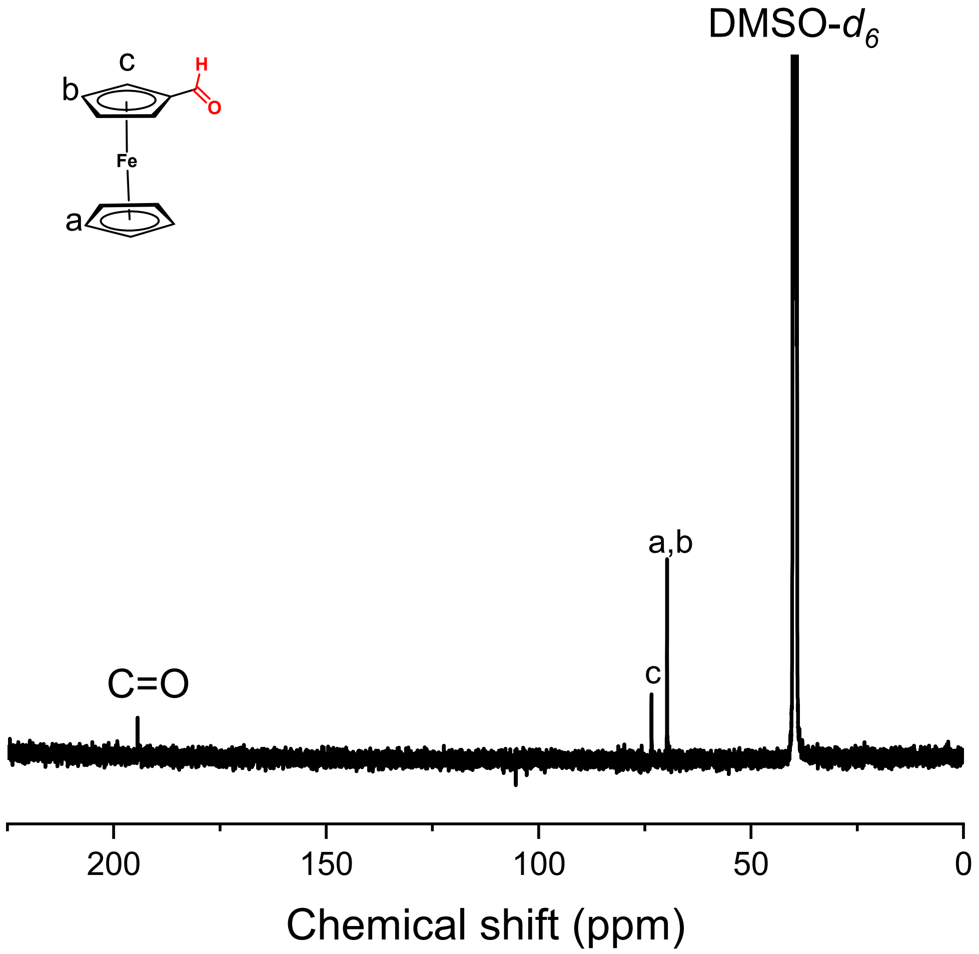

Figure S3. ^{13}C-NMR spectrum of FEC-CHO.

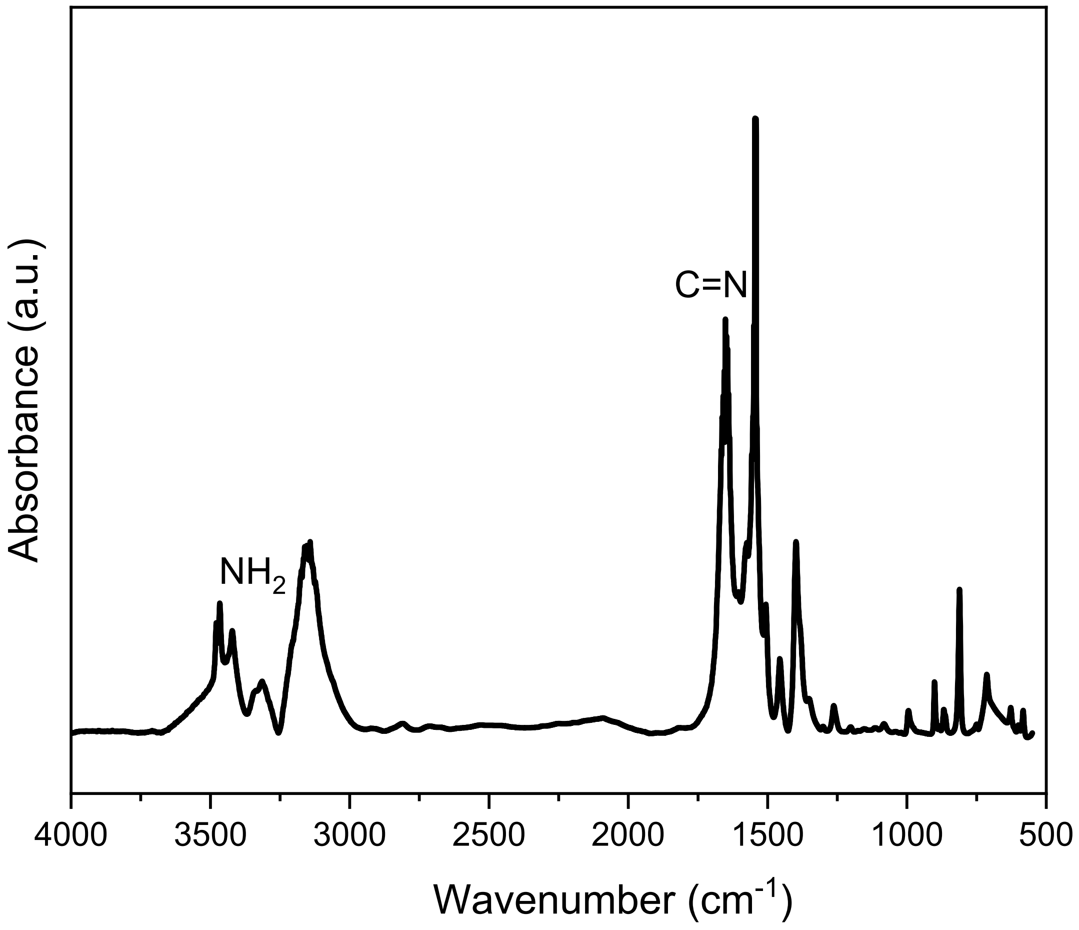

Figure S4. FTIR spectrum of PBDT.

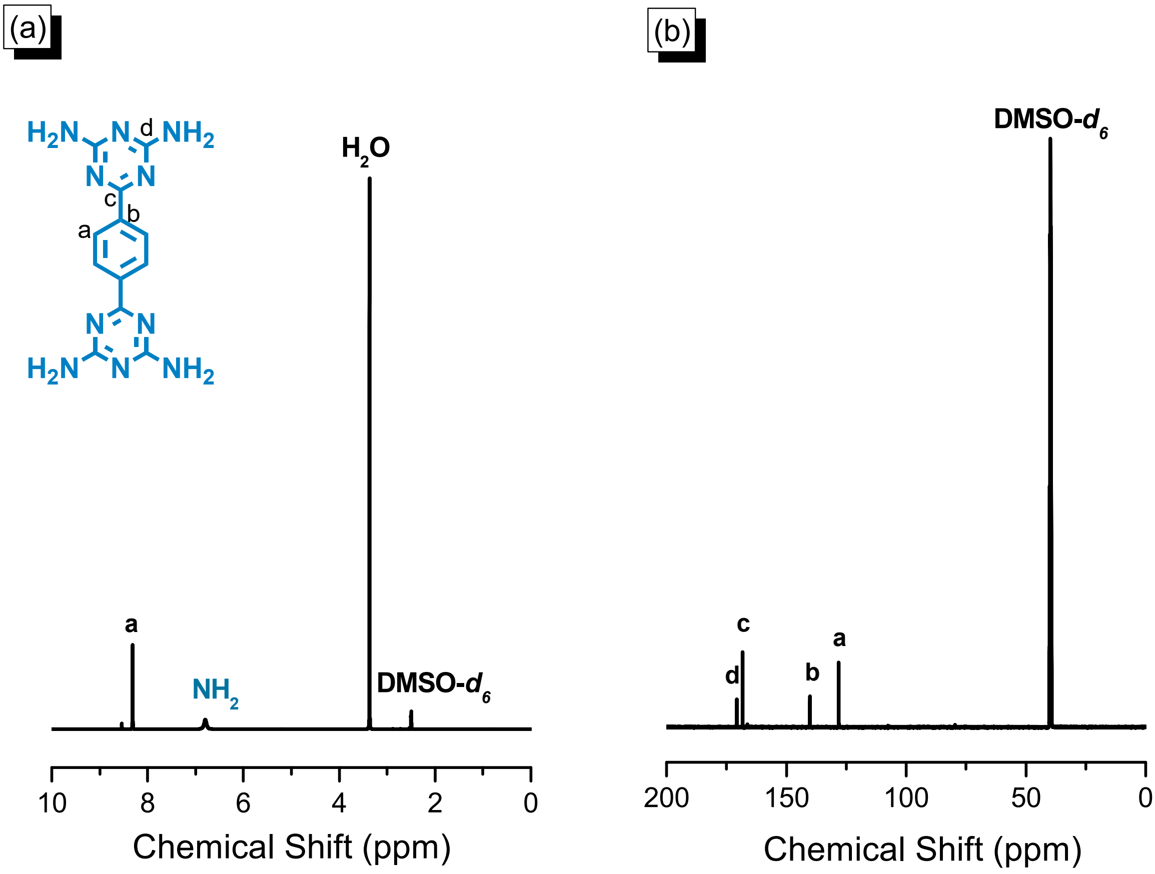

Figure S5. (a) ^{1}H-NMR and (b) ^{13}C-NMR spectrum of PBDT.

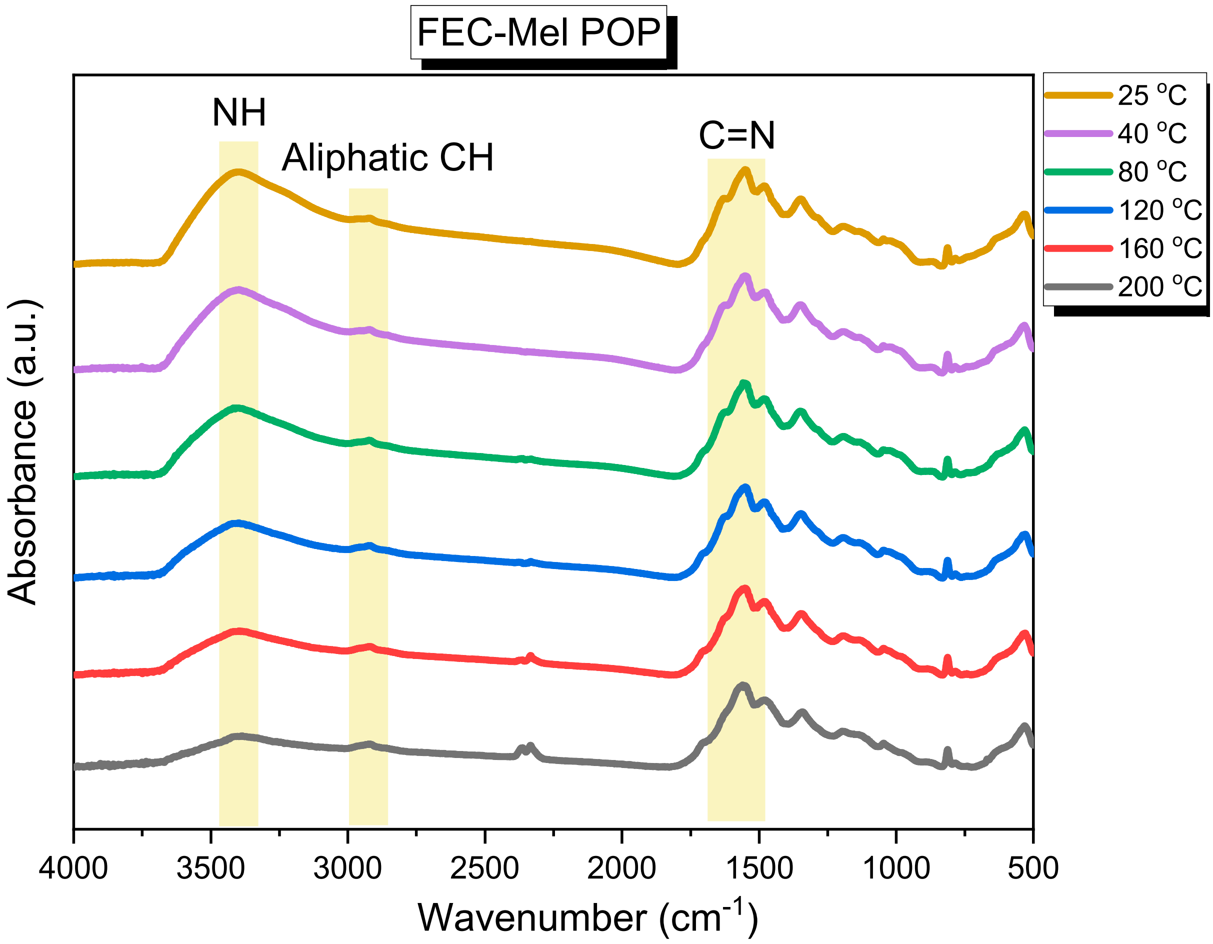

Figure S6. Corresponding FTIR profiles of FEC-Mel POP, measured at various temperatures (from 25 to 200 °C).

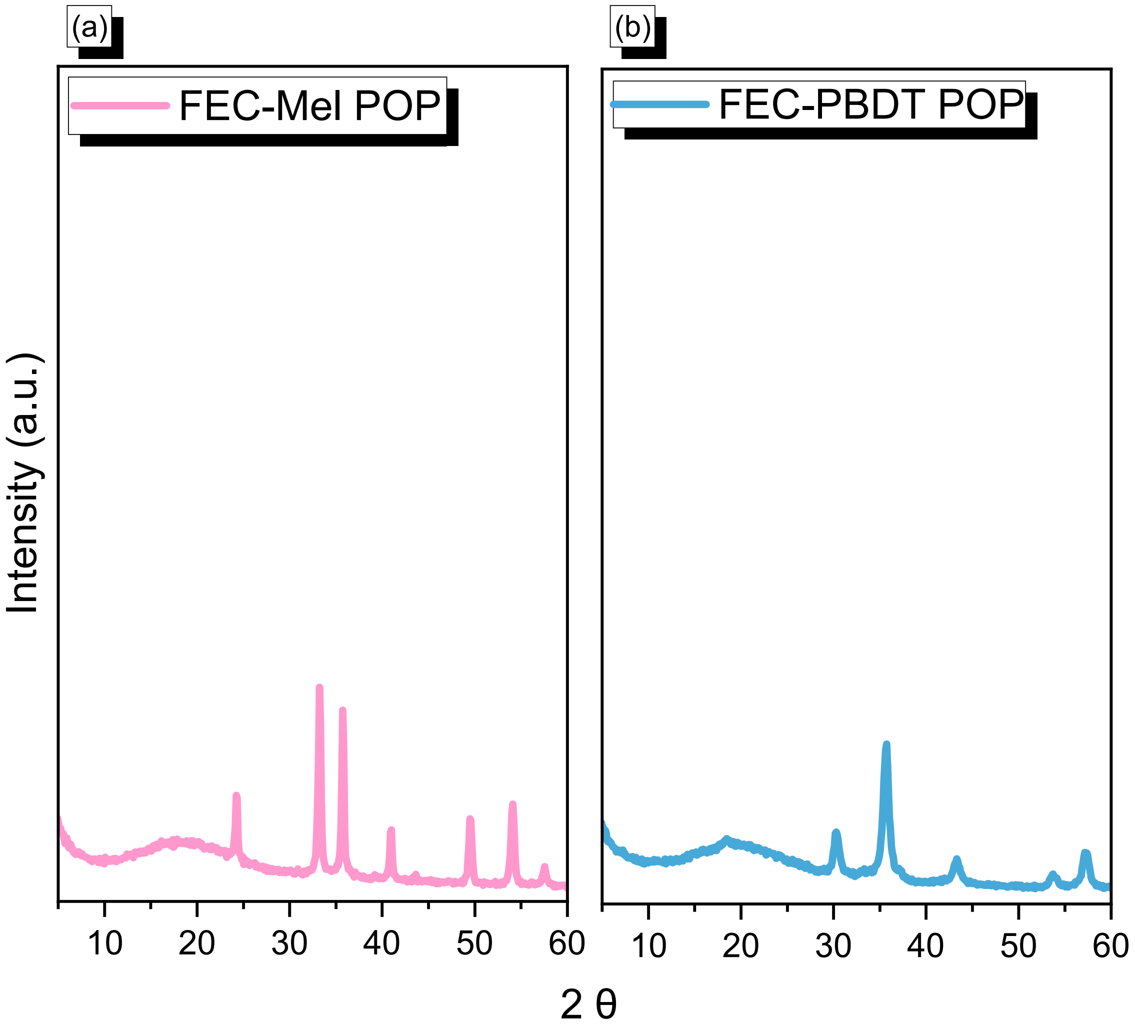

Figure S7. Corresponding XRD profiles of (a) FEC-Mel and (b) FEC-PBDT POPs.

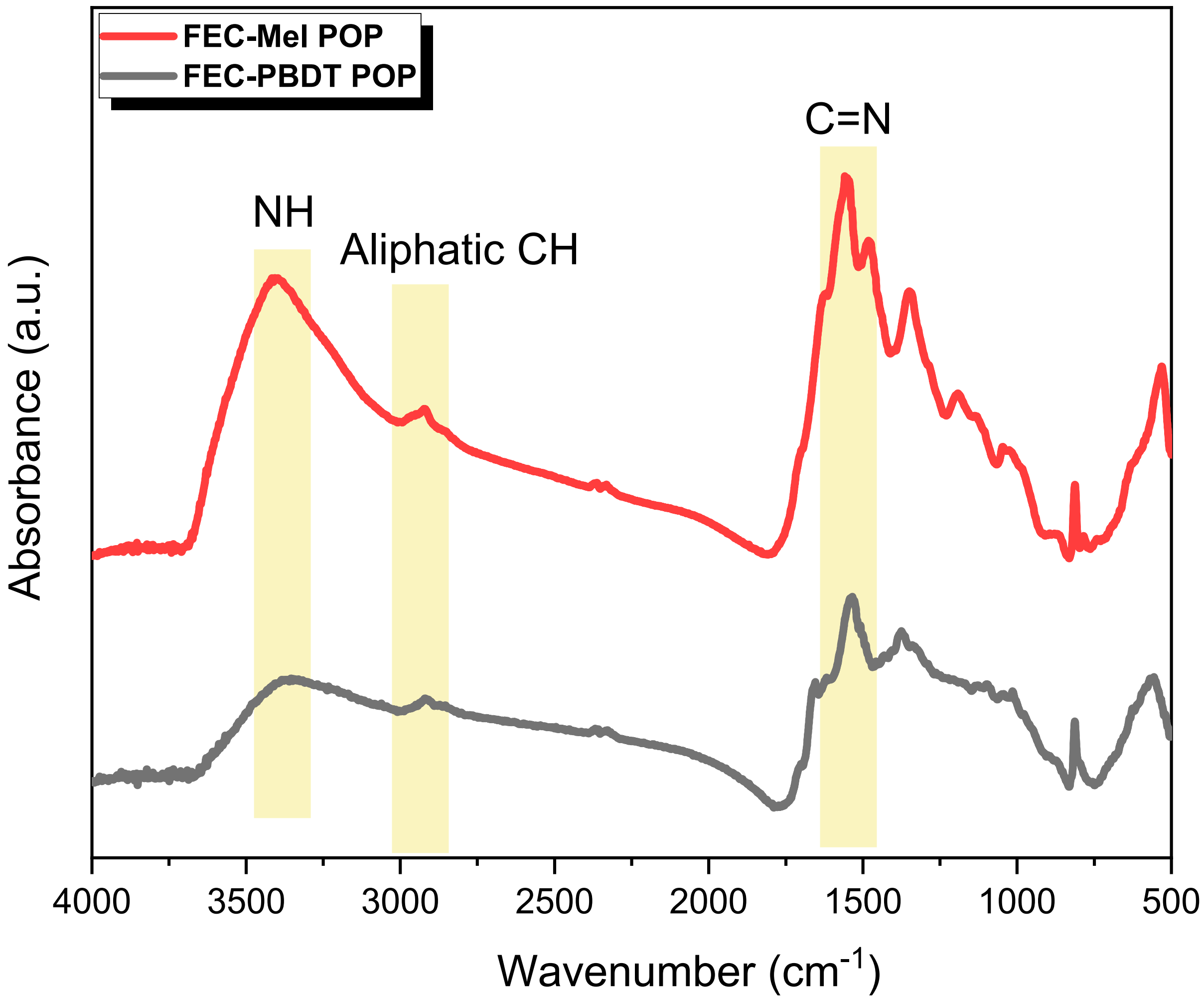

Figure S8. Corresponding FTIR profiles of FEC-Mel and FEC-PBDT POPs, recorded after electrochemical analyses.
